# A rare case of metachronous triple cancers involving the tympanic membrane

**DOI:** 10.12669/pjms.291.2490

**Published:** 2013

**Authors:** Hui-Yu Chang, Hua Jiang, Fang Zhou

**Affiliations:** 1 Hui-Yu Chang, MD, Department of Otolaryngology, 2nd Affiliated Hospital, School of Medicine, Zhejiang University, Zhejiang, China.; 2 Hua Jiang, MD, PhD, Department of Otolaryngology, 2nd Affiliated Hospital, School of Medicine, Zhejiang University, Zhejiang, China.; 3 Fang Zhou, MD, Department of Pathology, 2nd Affiliated Hospital, School of Medicine, Zhejiang University, Zhejiang, China.

**Keywords:** Metachronous triple cancers, Adenoid cystic carcinoma, Squamous cell carcinoma, Maxillary sinus, Tympanic membrane

## Abstract

Multiple primary malignancies are not rare. While metachronous triple cancers are rare and a triple tumor case involving maxillary sinus and tympanic membrane is exceptionally rare. We present such an extremely rare case with the index tumor of adenoid cystic carcinoma of the maxillary sinus and 14 years later esophageal cancer was observed as a metachronous tumor. One year after esophageal cancer, squamous cell carcinoma arising from tympanic membrane was detected. Before the tumor of tympanic membrane was observed, the patient had received total three radiation courses. Prior radiation therapy is suspected to be playing a role in inducing the squamous cell carcinoma of the tympanic membrane.

## Introduction

 Multiple primary malignancies are not rare. However a case involving the tympanic membrane is very rare. We present such an extremely rare triple primary tumor case with the index tumor of adenoid cystic carcinoma (ACC) of maxillary sinus and two metachronous tumors of squamous cell carcinomas (SCC) of tympanic membrane and esophagus.

## Case Report

**Fig.1:**
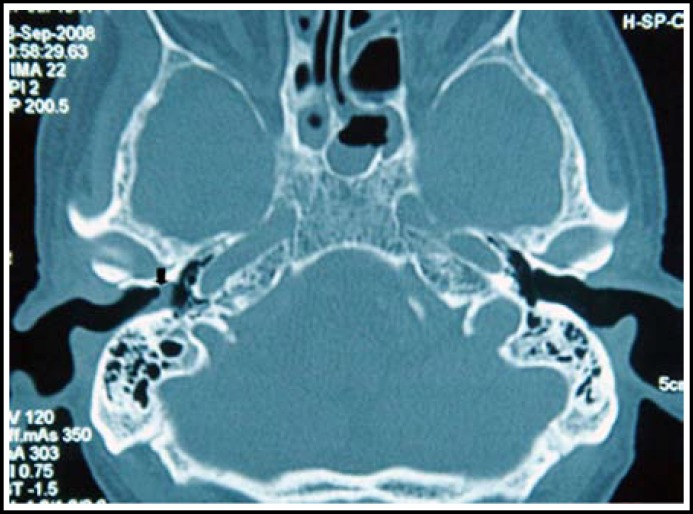
Computed tomography scan of the temporal bone. The tumorous mass is indicated by an arrow

 A 67-year-old man presented at our Department in September 2008 with a history of purulent ear drainage for three months. Examination revealed a tumor mass arising from tympanic membrane in the right external auditory canal. A computed tomography (CT) examination showed the tympanic cavity and the bone of the external auditory canal were not involved (Fig.1). The histopathological diagnosis of the specimen obtained from a biopsy was squamous papilloma with mild and moderate dysplasia. Subsequently, a tympanotomy was performed and the tumor was removed completely. Postoperatively, malignant change of the papilloma was found by histologic examination and a diagnosis of SCC was made. All surgical margins were negative. Clinical examination showed no SCC existed elsewhere. The suggested adjuvant radiation therapy was refused by the patient.

 During the postoperative course, no signs of either local tumor recurrence or distant metastases were found on review at twenty-two months. However, it is the second metachronous tumor in the patient.

 In August 2007, the first metachronous tumor in the patient was confirmed. Because of dysphagia, barium esophagogram was performed and neoplasm located in the thoracic esophagus was detected. Gastroscopic examination was then performed and tumor mass was found. A diagnosis of SCC was confirmed by biopsy. For treatment, the patient received 60 Gy of radiation alone and the tumor completely regressed. During 18 months follow-up, he remained free of symptoms and complaints, and no recurrence or distant metastases were detected.

 The index tumor was ACC of the maxillary sinus. At the age of 52, total maxillectomy was performed for the diagnosis of ACC of the right maxillary sinus. The patient then received postoperative radiation therapy at the dose of 66 Gy. Six months later, local recurrence was found and the patient underwent 65 Gy radiation treatment. After that, during the 15-year follow-up period, no recurrence or metastases were observed.

## Discussion

 Most primary malignant tumors arising in the paranasal sinus are SCC. ACC is the second most common malignancy and accounts for 5% to 15% of all paranasal sinus cancers.^1^ The maxillary sinus is the most common site.^2^^,^^3^ Because of late detection owing to sinusitis-like symptoms and relative surgical inaccessibility owing to important adjacent structures, maxillary ACC is recognized to have a unfavorable prognosis.^2^^,^^3^ Many studies have indicated that surgery with postoperative adjuvant radiation therapy is the main strategy for sinonasal ACC.^2^^-^^4^ However, patients who were treated with surgery and postoperative radiation had a high recurrence rate of 65%.^2^ In our case, local recurrence six months after initial surgery and radiation treatment was observed. This may be due to perineural invasion in ACC.^2^^,^^5^

 Unlike other malignancies in head and neck, to our knowledge, multiple primary malignancies with sinonasal ACC have not been reported before, which may be partly owing to the low incidence of sinonasal ACC. In this patient, maxillary ACC was the index tumor and 14 years later esophageal cancer (EC) was observed as a metachronous tumor. One year after EC, SCC arising from tympanic membrane was detected.

 SCC of the tympanic membrane is extremely rare and appearing in the patient with sinonasal ACC has not to our knowledge been reported previously. As the pathological examination indicated malignant change of the papilloma and clinical examination showed no SCC existed elsewhere, we believed that this tumor is independent of the others. However, it may be partly attributed to prior radiation. The tympanic membrane is in the maxillary radiotherapy field in this patient. And radiation therapy is regard as a risk factor of SCC of this site.^6^ In our patient, total three radiation courses were performed. It may be a risk factor for malignant transformation.

 SCC has a more aggressive behavior and a worse prognosis than other tumors of the auditory canal and middle ear.^7^ The outcome is related to the stage of the tumor. Local recurrence and distant metastasis were 16% in patients with SCC of middle ear.^7^ The therapeutic strategy for these patients is a complete removal of the tumor with free margins followed by postoperative adjuvant radiation.^8^^,^^9^ However, in Lobo D’s study, significant differences have not been found between the patients treated with surgery and radiation and with surgery alone.^7^ In our case, the patient received surgery alone and the surgical margins were free. No local recurrence or distant metastases were detected during twenty-two months of follow-up.

 In conclusion, we have presented an extremely rare case of multiple primary malignancies involving the tympanic membrane. Prior radiation therapy may play a role in inducing the SCC of the tympanic membrane. Our recent experience in treating such a patient seems important because it provided us with an opportunity to record this unusual case of multiple primary malignancies.
